# *Pseudomonas* Exotoxin Immunotoxins and Anti-Tumor Immunity: From Observations at the Patient’s Bedside to Evaluation in Preclinical Models

**DOI:** 10.3390/toxins11010020

**Published:** 2019-01-05

**Authors:** Yasmin Leshem, Ira Pastan

**Affiliations:** 1 Laboratory of Molecular Biology, Center for Cancer Research, National Cancer Institute, National Institutes of Health, Bethesda, MD 20892-4264, USA; jasminleshem@gmail.com; 2 Laboratory of Molecular Immunology, Faculty of Biology and Technion Integrated Cancer Center, Technion-Israel Institute of Technology, Haifa 3200003, Israel

**Keywords:** immunotoxin, anti-tumor immunity, immunotherapy, *Pseudomonas* exotoxin A, anti-PD-1

## Abstract

Immunotoxins are protein drugs composed of a targeting domain genetically fused to a protein toxin. One killing domain being explored is a truncated *Pseudomonas* exotoxin A (PE). PE based immunotoxins are designed to kill cells directly by inhibiting their ability to synthesize proteins. However, observations from clinical trials suggest that this alone cannot explain their anti-tumor activity. Here we discuss patterns of clinical responses suggesting that PE immunotoxins can provoke anti-tumor immunity, and review murine models that further support this ability. In addition, we describe our preclinical effort to develop a combination therapy of local PE immunotoxins with a systemic anti-CTLA-4 immune check point blocking antibody. The combination eradicated murine tumors and prolonged the survival of mice. Clinical trials that test the ability of immunotoxins to augment immunotherapy have been recently opened.

## 1. Introduction

The development of immunotoxins as cancer therapeutics was based on the rationale that toxins evolved in nature to become highly cytotoxic. Immunotoxin mechanisms of action were studied in cell culture, and also in tumors growing in immune compromised animals without taking into account possible interactions with the immune system [1]. Thus, effects on the immune system were only discovered at a later stage, when immunotoxins reached clinical trials. One major finding discovered in patients was that immunotoxins lead to the formation of neutralizing anti-drug antibodies (ADAs). This aspect of immune activation has been extensively researched, and efforts to overcome this obstacle are ongoing [2,3]. However, the ability of immunotoxins to elicit a strong immune response might also have a positive aspect. The data reviewed here suggest that they induce anti-tumor immunity and can act as a mode of immunotherapy. Perhaps both ADAs and anti-tumor immunity are the result of the immunotoxin’s ability to provoke a strong immune activation and thus represents two sides of the same coin.

## 2. PE Based Immunotoxins

Immunotoxins are protein drugs that contain a cell killing domain genetically fused to a targeting moiety [4,5,6]. *Pseudomonas exotoxin A* (PE) is one toxin that is being used as the immunotoxin killing moiety. PE catalyzes the transfer of ADP ribose from nicotinamide adenine dinucleotide (NAD) to elongation factor 2 (EF2) [7]. This transfer irreversibly modifies EF2, inactivates it and leads to protein translation arrest and cell death by apoptosis [8,9,10]. A few clinical trials have evaluated PE based immunotoxins in humans [11]. Of these, clinical success was best achieved with Moxetumomab pasudotox for the treatment of hairy cell leukemia. Moxetumomab pasudotox contains a truncated PE of 38 kDa in size (PE38), that is fused by genetic engineering to a portion of an anti-CD22 antibody. In a pivotal trial evaluating 80 patients with relapsed/refractory hairy cell leukemia treated with Moxetumomab pasudotox, the complete response rate was 41% and the overall response rate was 75%. Complete response was usually noted during therapy. However, in five patients, a delayed complete response was noted six months after the end of treatment, suggesting that in some patients the toxin does more than directly kill cells. This trial led to FDA approval of Moxetumomab pasudotox in 2018 [12,13]. Achieving significant tumor regressions in common solid tumors using PE based immunotoxins has been more challenging, because patients with solid tumors have a functioning immune system that allows them to develop ADAs which neutralize immunotoxin activity [2].

## 3. Inhibition of Protein Synthesis as a Possible Trigger of Immunity

Protein synthesis is a fundamental process in living cells. Some pathogens produce toxins that reduce protein synthesis in the host. Examples are the PE of *Pseudomonas aeruginosa*, several toxins made by *Legionella pneumophila*, Shiga toxin and toxins made by *Staphylococcus* and shigatoxigenic serotypes of *Escherichia coli* [10,14,15]. Some researchers have suggested that inhibition of protein synthesis is a pathogen-associated damage pattern that promotes immunity. For example, inhibition of protein synthesis by PE can induce a gene transcription program in Caenorhabditis elegans (*C. elegans*) that prevents its mortality. A similar transcription program is initiated in *C. elegans* in response to other agents that reduce protein synthesis, but not to a mutated inactive PE [16,17]. In addition, exposure of macrophages to *L. pneumophiia* containing virulent factors that inhibit protein synthesis results in a specific transcriptional program and recruitment of host cells to the infection site. The transcriptional changes include activation of nuclear factor-kappa B (NF-κB) and a mitogen-activated protein kinase (MAPK), and can be recapitulated by other insults capable of reducing protein synthesis, including exposure to PE [18,19].

## 4. The Restrained Power of Anti-Tumor Immunity

The immune system often recognizes tumors but cannot efficiently execute an immune attack [20]. A paradigm shift in oncology occurred when antibodies blocking the immunological checkpoint cytotoxic T-lymphocyte-associated antigen 4 (CTLA-4) caused disease regression in melanoma patients [21] and, even more so, when antibodies that block interactions between programmed cell death protein 1 (PD-1) and its ligands caused disease regression in patients with melanoma [22], non-small-cell lung cancer [23], renal cell carcinoma [24], head and neck squamous cell carcinoma [25], urothelial carcinoma [26], Mismatch repair-deficient (dMMR) tumors [27], Hodgkin’s lymphoma [28] and Merkel-cell carcinoma [29]. These successes established immunotherapy as a major treatment modality in oncology. However, most patients still do not respond to immune checkpoint blocking agents [30]. The current effort in the field is focused on elucidating ways to increase both the percentage of responding patients and the diversity of tumor entities that can be successfully treated with immune checkpoint blockers [31].

## 5. Clinical Observations Suggesting that PE Immunotoxins Induce Anti-Tumor Immunity

Across clinical testing of different PE based immunotoxins, isolated cases in which unexpected patterns of tumor regressions were noted. These include slow or delayed tumor regression, maintenance of tumor control after the patients were off treatment, pseudo-progression, and discordance between tumor regression and increased signal captured by positron emission tomography–computed tomography (PET-CT). We suggest that anti-tumor immunity plays a role in these patterns of clinical effects. Cytotoxic drugs, such as immunotoxins, are expected to kill cells only when they are actively present in the patient’s body. These patterns of tumor regressions might represent the induction of anti-tumor immunity that once initiated, remains active after the cytotoxic drug has been cleared. Similar patterns of responses have been described in patients receiving immune check point blockers [32,33,34]. Because PET monitors the increase in metabolic activity, a positive signal could represent infiltration of immune cells and Could explain an increased signal in PET scan during tumor regression [35,36,37]. In this section we summarize the clinical data supporting the hypothesis that PE immunotoxins induce anti-tumor immunity.

### 5.1. Systemically Administered SS1P in Combination with Immune Modulating Chemotherapies

**Figure 1:**
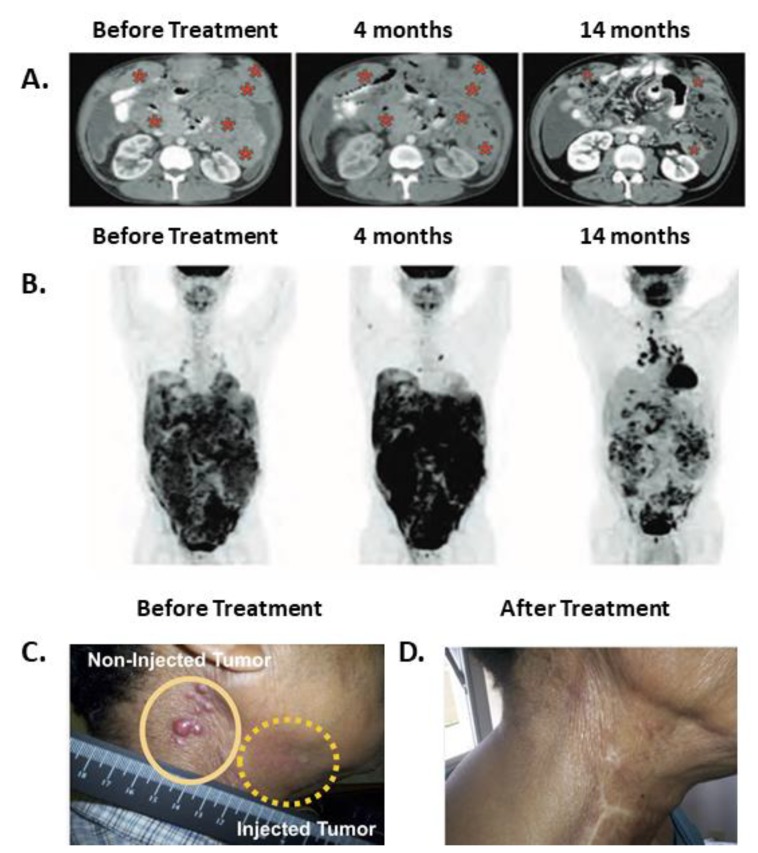
Regression patterns after immunotoxin therapy suggest that anti-tumor immunity developed. (**A**,**B**) A patient follow-up after receiving IV SS1P, pentostatin and cyclophosphamide. Abdominal CT (**A**) and PET scans (**B**) before treatment, at 4 months, and at 14 months from entering the trial. Red asterisks indicate tumor mass. This patient completed two cycles of treatment and received no treatment thereafter. The figure was reproduced from Hassan et al. 2013, with permission from science publishing group [38]. (**C,D**) Resolution of injected **(C)** and non-injected **(D)** tumors in a patient with squamous cell carcinoma of the head and neck. VB4-845 immunotoxin was injected only into the tumor marked with a dashed yellow line. The figure was reproduced from MacDonald et al. [39]. 2009, and modified with permission from Dove Medical Press.

SS1P is a PE38 based immunotoxin that targets mesothelin. A clinical trial evaluated the effects of SS1P in combination with immune modulating chemotherapies in mesothelioma patients [38]. The rationale underlying the use of this drug combination was to modulate the immune system by using pentostatin and cyclophosphamide to reduce the production of neutralizing antibodies formed against SS1P. Each treatment cycle lasted 21 to 30 days. Of the 10 patients who received the treatment, three had durable partial responses lasting after the trial drugs were discontinued, suggesting that anti-tumor immunity had a role in the anti-tumor effects. One responding patient demonstrated shortness of breath at her first cycle, and a CT scan performed during that incident revealed marked tumor progression. A second scan performed 2 months later showed tumor regression. Tumor control was maintained (up to a 74% reduction in size) during and after receiving six treatment cycles up to her last reported visit at 15 months. A similar pattern of short-term disease progression followed by long lasting disease regression was described after anti-CTLA-4 or anti-PD-1 therapy and has been termed pseudo-progression [34]. The second responding patient received four treatment cycles until he developed ADAs. His PET scan at 1.6 months showed a marked increase in signal which was discordant with a CT scan performed at the same time that showed disease regression. At subsequent follow-up, the PET scan signal completely disappeared, aligning with the CT scan that continued to show tumor regression of up to 70% of the original tumor mass. The third responding patient received only two treatment cycles. His first and second CT scans performed at 2 and 4 months demonstrated stable disease. As with the previously described patient, there was discordance at 4 months between the increased signal shown on the PET and CT scan results. Although this patient no longer received treatment, his tumor continued to regress and by 7 months reached the qualification for partial response according to the response evaluation criteria in solid tumors (RECIST). At this point, signal reduction was also noted with the PET scan aligning with the CT scans. This patient maintained a partial response until his last reported visit at 15 months. Representative images of this patient follow-up are shown in Figure 1A,B. In addition, two patients who were unresponsive to chemotherapy before the trial, became chemo-sensitive after the SS1P treatment.

### 5.2. Intra-Tumoral PE Immunotoxins in Patients with Epithelial Cancers

A phase I clinical trial evaluated the safety of intra-tumor injection of VB4-845, an immunotoxin targeting epithelial cell adhesion molecule (EpCam), in 20 patients with squamous cell carcinoma of the head and neck (SCCHN) [39]. The compound was injected directly into the tumors once a week. Of the 20 injected tumors, four completely regressed, six partially regressed and four stabilized. The most frequent side effects were injection site edema, redness, and pain (tumor, rubor, and dolor). All can be grouped together as the cardinal signs of inflammation. The fourth classic sign of inflammation is local heat (calor) [40]. Though local heat was not reported, systemic fever was noted in four of 20 patients. In addition, regression of un-injected tumor site was noted in three patients. A photograph of a representative patient is shown in Figure 1C. Regression of un-injected tumors can be attributed either to a direct cytotoxic effect of VB4-845 immunotoxin that leaked from the local injection site to the systemic circulation, or it can be a result of an indirect effect achieved by anti-tumor immunity. Because use of IV anti-EpCAM immunotoxin did not result in tumor regressions in a separate study [41], we suggest that anti-tumor immunity is more likely to explain regressions of un-injected tumors in this study.

In another study, 11 patients with cutaneous tumors received intra-tumor scFv (FRP5)-ETA immunotoxin [42]. This compound targets ErbB2 (HER2/Neu). All patients received daily treatment for 7 to 10 days. Complete regression of injected tumors was reported in four patients, and partial regression in three. There were no reports of regression of non-injected tumors. The most common side effects were injected site pain and inflammation and were documented in 9 out of 11 patients. Criteria for inflammation were not defined in this study.

### 5.3. Intra-Tumoral PE Immunotoxins to Treat Brain Tumors

The brain is protected from the systemic circulation by the blood-brain barrier, making it extremely difficult for protein drugs to penetrate [43]. One strategy for improving drug delivery is to administer the drug as a localized treatment directly into the brain. Several clinical trials have been carried out to evaluate the use of locally delivered PE38 immunotoxins for the treatment of brain tumors. We will summarize two trials in which some patients were reported to have slow or delayed tumor regression.

A phase I clinical trial evaluated the use of NBI-3001 administered locally into 31 patients with recurrent malignant glioma [44]. NBI-3001 is an IL-4-PE38KDEL immunotoxin that binds to IL-4 receptor overexpressed by some brain tumors. NBI-3001 was infused through a catheter directly into the tumors and was given once to each patient. The authors noted that immediately after the infusion there were distinct regions of decreased signal in MRI. This might represent necrotic regions. However, for other regions in the tumors, reduction of pathological signals took weeks to months to become apparent. Two patients with long-term survival from this trial were further described. One patient with glioblastoma multiforme (GBM) underwent gross tumor resection 3 months after the treatment and remained disease free 3 years later. In another GBM patient some tumor regression was observed at his first checkup 4 weeks after the treatment. His tumor had continued to regress, with maximal regression seen a few months later, by which time it decreased to 5% of the original tumor volume. This patient remained stable for 3 years until his cancer regrew and led to his death [45]. To note, GBM is very aggressive, and long-term survival is rare [46,47].

In another phase I clinical trial, local administration of TP-38 immunotoxin was evaluated in patients with brain tumor recurrences [48,49,50]. TP-38 is an immunotoxin composed of truncated PE38 fused to transforming growth factor alpha (TGF-α) and targets epidermal growth factor receptor (EGFR). TP-38 was infused through a catheter into the brain over a period of 50 h. Of the 20 patient-cohort, 15 had residual disease at the time of treatment. In this group, two patients with GBM experienced disease regression. One patient had a gradual decrease in the pathological enhancement signal up to near-complete remission. This patient was reported alive at 198 weeks. The second patient had a gradual and sustained partial response with disease shrinkage of >50% at 24 weeks. His death at 34 weeks was reported as unrelated to his brain tumor. Because a direct effect of the drug was only expected to occur near the treatment period, we suggest that these patterns of gradual tumor regression are a result of anti-tumor immunity. Of the five patients in this trial who had no residual disease at the time of treatment, one was disease-free at 211 weeks from therapy. This patient had developed a large area of contrast enhancement 9 weeks after therapy that later regressed completely. Because that region was never biopsied, it remains unclear whether that event had been a result of inflammation or, alternatively, of disease progression that had spontaneously regressed. Table 1 summarizes the clinical observations which suggest that PE immunotoxins induce anti-tumor immunity.

## 6. Anti-Tumor Immunity Achieved by PE Immunotoxins in Preclinical Murine Models

Ochiai et al. examined the effect of EGFRvIII-targeted MR1-1 immunotoxin in a model of murine astrocytoma [51]. They found that when SMA560 EGFRvIII cells were injected together with MR1-1 immunotoxin, cells did not form tumors. This effect was abolished, however, when the same conditions were tested in mice depleted of either CD4 or CD8 cells, indicating that the effect depends on the immune system. In addition, long-term anti-tumor immunity protected those mice from a second challenge with the same cells. Another group evaluated the ability of the IL13-PE38 immunotoxin to promote anti-tumor immunity in small established tumors. In this study they used D5 murine melanoma cells transfected with human IL13Rα2 (D5 IL13Rα2) and inoculated the cells in two different locations [52]. They found that injecting IL13-PE38 immunotoxin into D5 IL13Rα2 tumors, but not into D5 tumors, slowed the growth rate of D5 IL13Rα2 tumors that were growing on the same mice but were not injected. This growth inhibition was blocked by depletion of CD4 and CD8 positive cells, indicating that the effect was mediated by the immune system. In addition, injection of IL13-PE38 immunotoxin was associated with an increase in CD4 and CD8 positive cells in both injected and non-injected tumors.

## 7. Synergy between Local Anti-Mesothelin Immunotoxins and Systemic Anti-CTLA-4 in Murine Cancer Models

One possible way to use immunotoxins as immunotherapy is to combine them with an immune checkpoint inhibitor. We evaluated the combination of locally delivered anti-mesothelin immunotoxins and systemic anti-CTLA-4 in both the 66C14-M murine breast cancer model and the AE17-M murine mesothelioma model. In the breast cancer model, treatment with anti-CTLA-4 given IP and intra-tumor (IT) SS1P injected into one of two tumors growing in the same mouse resulted in complete regressions in 86% of the injected tumors and 53% of non-injected tumors. No tumor regression was demonstrated in mice that were given each drug alone. These findings indicate that both synergistic and systemic effects were achieved by this therapy. The anti-tumor effect was associated with increased tumor CD8 positive cells and was dependent on the presence of this cell population [53]. Furthermore, in the cured mice new 66C14 tumors not expressing human mesothelin were rejected, indicating that long-term anti-tumor immunity was formed. In the AE17-M mesothelioma model we found that exposure of cells to SS1P induced surface calreticulin expression and elicited ATP secretion, both of which are markers for immunogenic cell death. In addition, the combination of IT SS1P and IP anti-CTLA-4 significantly prolonged the survival of AE17-M tumor-bearing mice compared to control groups [54].

One possible limitation of the combined administration of an immunotoxin and an immune modulator is a possible increase in ADAs. Mazor et al. showed that the formation of ADAs to anti-mesothelin immunotoxin was increased when the immunotoxin was combined with anti-CTLA-4 or anti-OX40 antibodies. No increase in ADAs was demonstrated, however, when an immunotoxin was combined with anti-PD-1 or anti-PD-L1 antibodies [55]. Combining anti-CTLA-4 and anti-mesothelin immunotoxin with tolerogenic nanoparticles that contain rapamycin was found to reduce ADAs formation [56]. Table 2 summarizes the pre-clinical models showing evidence of anti-tumor immunity with immunotoxin treatment.

## 8. Concluding Remarks

Observations from the bedside of patients led us and others to explore the ability of immunotoxins to induce anti-tumor immunity in preclinical models. In murine models it was verified that immunotoxins can induce anti-tumor immunity and can be used locally to prime tumors to an immune attack elicited by anti-CTLA-4. Some substantial questions remain open. For example, what is the mechanism of action, and whether other immune checkpoint inhibitors such as anti-PD1 will prove to be good candidates for combined administration with immunotoxins. Clinical trials aimed at answering some of these questions were recently opened (NCT03258593, NCT03644550) and their results are pending.

## Figures and Tables

**Table 1:** Clinical Data Suggesting that PE Immunotoxins Provoke Anti-Tumor Immunity.

Cancer Type	Drug and References	Target	Route	Other Drugs	Patients (*n*)	Clinical Effect	Delayed Effect	Findings in Support of Anti-Tumor Immunity
Hairy cell leukemia	Moxetumomab Pasudotox [12]	CD22	IV	None	80	41% CR, 75% OR	5 pts	Delayed clinical effects
Mesothelioma	SS1P [38]	MSLN	IV	CP + P	10	0 CR, 3 PR, 3 SD, 4 PD	2 pts	Delayed clinical effects; maintenance of tumor control; disease regression accompanied by an increased signal on PET-CT.
SCCHN	VB4-845 [39]	Ep-Cam	IT	None	20	Injected site: 4 CR, 6 PR, 4 SD	Not reported	Local redness, edema and pain. Systemic fever in 4 patients; three patients showed regressions of non-injected tumor sites.
Cutaneous metastases	ScFv(FRP5)-ETA [42]	ErbB2	IT	None	11	Injected site: 4 CR, 3 PR	Not reported	Local inflammation at injection site (symptoms not specified); systemic fever in 2 patients.
Brain tumors	TP-38 [49,50]	EGFR	IT	None	15 residual disease	1 near CR, 1 durable PR	2 pts	Slowly occurring clinical effects; maintenance of tumor control.
					5 NED	4 PD, 1 NED	N/A	A case of contrast enhanced area that appeared at 9 weeks and then disappeared spontaneously.
Brain tumors	NBI-3001 [44,45]	IL-4 R	IT	None	31	Radiographic signs of tumor necrosis in 71% of pts	Not reported	Typically, MRI contrast enhancement decreased after the infusion, then increased at 4 weeks, and then again decreased gradually. One patient had durable partial regression lasting for 3 years.

*n*, number of patients; pts, patients; IT, intra-tumoral; IV, intravenous; CP, cyclophosphamide; P, pentostatin; CR, complete response; OR, overall response rate; PR, partial response; SD, stable disease; PD, progressive disease; SCCHN, squamous cell carcinoma of the head and neck; NED, no evidence of disease; N/A, not applicable.

**Table 2:** Lessons Learned in Preclinical Models.

Effect	Contributing Evidence	Murine Models	Toxins	Ref. #
Anti-tumor effect of immunotoxins is partially mediated by the immune system	Depletion of CD8+ cells with or without depletion of CD4+ cells reduces survival	Malignant astrocytoma, Melanoma	MR1-1, IL13-PE38	[51,52]
Immunotoxins render tumors more sensitive to immune checkpoint inhibitors	Increased survival and number of tumor infiltrating CD8+ cells demonstrated in the combination treated mice.	Breast cancer, Mesothelioma, Glioma	SS1P, LMB-100, D2C7	[53,54,57]
Immunotoxins induce markers of immunogenic cell death	Increased ATP secretion and calreticulin surface expression.	Mesothelioma	SS1P, LMB-100	[54]
